# Anemia and transfusion of red blood cells

**Published:** 2013-12-31

**Authors:** Armando Cortés Buelvas

**Affiliations:** Pathology Department in the School of Health, Universidad del Valle E-mail address: acortes59@gmail.com

**Keywords:** anaemia, blood transfusion, transfusion practice

## Abstract

The red cells transfusion is a mainstay in the treatment of anemic patients. These blood transfusions are not without risks. The risk-benefit profile for red cell transfusions to treat anaemia is uncertain, but they may contribute to adverse patient outcomes in some situations. The ability of a patient to tolerate anaemia depends on their clinical condition and the presence of any significant co-morbidity; maintenance of circulating volume is of paramount importance. There is no universal transfusion trigger. Advances in the development and validation of physiological, accessible, practical and reliable markers to guide therapy are expected. To improve patients' outcomes, further study is required to more fully explore the risk of anemia, optimal hemoglobin level, and the risk and efficacy of RBC transfusion. Future clinical investigations with high priority should determine the efficacy of transfusion in those classified as uncertain scenarios. In the absence of data, it is prudent that transfusion is administered with caution in these clinical scenarios.

## Abstract (es)

La transfusión de glóbulos rojos es uno de los pilares en el tratamiento de pacientes anémicos. Las transfusiones de sangre no están libres de riesgo. Aunque es incierta la relación riesgo-beneficio de las transfusiones para tratar la anemia, estas pueden ocasionar resultados adversos de los pacientes en algunas situaciones. La capacidad de un paciente para tolerar la anemia depende de su estado clínico y la presencia de cualquier co-morbilidad significativa; la preservación del volumen circulante es de suma importancia. No existe un indicador automático universal para la transfusión. Se espera que los avances en el desarrollo y validación de marcadores fisiológicos, accesibles, prácticos y confiables permitan guiar la terapia. Para mejorar los resultados de los pacientes, se requieren más estudios que exploren más a fondo el riesgo de la anemia, el nivel de hemoglobina óptima, y el riesgo y la eficacia de la transfusión de glóbulos rojos. Las investigaciones clínicas futuras con alta prioridad deben determinar la eficacia de la transfusión en los clasificados como escenarios inciertos. Ante la falta de datos, es prudente que la transfusión se administre con precaución en estos escenarios clínicos.

## Introduction

Red blood cell (RBC) transfusions are a mainstay in the treatment of anemic patients, making it the most common medical procedure in hospitalized patients^1^.

Most RBC transfusions (RBCT) are prescribed for patients with relatively low levels of hemoglobin (Hb) and only in controlled situations. The underlying thinking is that the transfusion will increase oxygen transport and therefore decrease deficiencies thus "relieving" tissue hypoxia. However, this hypothetical benefit of RBC transfusions has not been unequivocally demonstrated.

An inadequate supply of oxygen to tissues can lead to multiple organ failure and increased morbidity and mortality. These deleterious effects appear only with very low Hb levels when compensatory mechanisms do not work properly or are insufficient; however, that level is not exactly known.

The risks and complications of RBCT include: the transmission of infectious diseases, immune suppression, acute respiratory distress syndrome, circulatory overload and errors in administration. With these potential or actual adverse effects taken together with the large variability in observed prescriptions, it has been determined that when faced with "liberal" or "defensive" traditional transfusion criteria, more restrictive transfusion practices are adopted along with an enhanced usage of alternative treatments. However, we must be cautious because this trend can lead us to expose patients unnecessarily to risks of anemia and hypoxia by not administering transfusions.

This makes it essential to specify an appropriate risk/benefit ratio for the transfusion. This is because it is also not permissible to subject the patient to an intervention whose effectiveness has not been documented in terms of reduced mortality or morbidity.

In the 1988 Consensus Conference of the NIH (National Institute of Health) the lack of justification for "classic" transfusion practice was highlighted and it was concluded that "the available evidence does not support the use of a single criterion for transfusion, such as Hb<100 g/L. No single measure can replace good clinical judgment as the basis for decision making concerning RBCT"^2^.

Weiskopf asserts that although millions of units of red blood cells are transfused, the biological effectiveness of this measure has not been demonstrated in prospective, controlled studies, nor do other criteria exist by which one could judge the effectiveness of the transfusion of concentrated red blood cells^3^ ^.^

After almost a century of the clinical use of RBCT, the continued use of this form of therapy is now being questioned. This is not necessarily due to adverse effects, but to the lack of studies that document its effectiveness.

A recent systematic review of the literature considered the results of 45 cohort studies with 272,596 patients in critical condition and revealed that in 42 of the 45 studies transfusion represented greater risk than benefit to patients and in the transfused cohort it presented a higher rate of adverse events than it did to those not receiving transfusions^4^.

Similarly, in clinical randomized controlled trials (RCTs) evaluating the efficacy/ effectiveness of RBCT^5^ ^-^ ^8^ it is indicated that the restriction of RBCT in patients without hemorrhage has no significant negative effect on patient outcomes, and it may even improve the results among some populations. Taken together, these studies suggest that RBCT provides minimal benefit for critically ill patients with hemoglobin (Hb) levels greater than 8-9 g/dL^9^ ^-^ ^12^. Additionally, it may contribute many undesirable results in many clinical situations, with increased morbidity, mortality and length of hospital stay^13^ ^-^ ^19^ ^.^

These findings oblige a more judicious use among the many patients who are routinely transfused. A greater effort should be made to understand that there is a transition point at which the physiological mechanisms fail to compensate for the decreased oxygen supply associated with anemia, in which case transfusion favors the results.

## Factors to consider in the analysis

It has been noticed that in young, alert, healthy adults when subjected to an isovolemichemodilution with a decreased hemoglobin concentration of 12.5 g/dL to 4.8 g/dL that an increased heart rate, systolic volume index and the cardiac index is produced with no systemic evidence of hypoxic changes despite a reduction in the DO_2_ (oxygen delivery) to a level of 7.3 mL/kg/min^20^.

A study conducted with septic patients suggests that the critical DO_2_ could be 3.8 mL/kg/ min^21^.

The decrease in DO_2_ with anemia does not always translate into decreased VO2 (oxygen consumption) due to the intervention of compensatory physiological mechanisms, such as the increase in cardiac output and tissue O_2 _extraction. The VO_2_ is, in turn, balanced by the ability of peripheral tissues to modify oxygen extraction (EO_2_) in hypoxemic states thus altering micro-vascular blood flow and maintaining a stable tissue pO_2_ ^22^.

The studies that reveal a lack of increased tissue O_2 _with RBCT have been interpreted as a lack in transfusion effectiveness and are attributed to the loss of 2,3-diphosphoglycerate and nitric oxide during storage or to increased viscosity^23^ ^,^ ^24^.

Cardiac function determines the clinical tolerance limit to anemia in any patient. Oxygen delivery to the myocardium increases only by means of improving the blood flow in the coronary arteries^25^.

The consequent reduction of coronary flow can thus increase the pressure at the end of the diastole, to produce changes in the ECG and subsequent symptomatic ischemia^26^. 

Some studies in humans have thrown light on the limits of physiological compensation of anemia. The critical Hb at rest has been estimated at approximately 20-25% of normal Hb^22^.

The first report was documented in an 84 year old Jehovah's Witness who refused transfusion and died after surgery with aHb of 1.6 g/dL. The critical DO_2_ in this patient under anesthesia was 4.9 mL O_2_/kg/min for a VO_2_ of 2.4 mL O_2_/kg/min and death occurred at aHb level of 4.0 g/dL. The dissociation curve of oxyhemoglobin, after correction for changes in the pH and PCO_2_, deviates to the right with a hematocrit of 8%. This indicates a decrease in oxygen affinity of hemoglobin as a compensatory mechanism to facilitate the delivery of O_2_ to peripheral tissues in extreme anemia^27^.

Furthermore, in critically ill patients sedated after cessation of life support, the DO_2_ varies between 3.8-4.5 mL O_2_/kg/min. For a VO_2_ of 2.4 mL O_2_/kg/min, the critical EO_2_ was approximately 60%^21^.

Considering that the critical DO_2_ varies with different metabolic requirements, subsequent studies were carried out taking measurements during states of consciousness in which the VO_2_ is higher. It has been shown that healthy humans at rest are able to tolerate acute isovolemichemodilution with a Hb of 5 g/dL^27^ -although a slight reduction in alertness occurs which is reversible^28^ ^,^ ^29^.

In 32 conscious individuals at rest, no significant change was produced any in the concentration of lactate or VO_2_ despite the decrease in DO_2_ during progressive isovolemichemodilution with 5% albumin and/or autologous plasma to a level of Hb 5 g/dL^30^.

In another study with conscious young healthy volunteers it was found that for a VO_2_ of 3.4 mL O_2_/kg/min, the critical DO_2_ during acute hemodilution with 5% albumin and autologous plasma was less than 7.3 mL O_2_/kg/min and 4.8 g/dL of Hb^20^.

However, in the presence of coronary artery disease, the Hb threshold may increase. In animal models it has been reported that Hb threshold may be at a level of 7-7.5 g/dL in the presence of coronary artery stenosis with limited tolerance to isovolemic hemodilution^31^ ^,^ ^32^.

Contractile dysfunction induced by ischemia and compromise in the delivery and consumption of O_2_ can be reversed and corrected with RBCT by increasing the Hb to 1.9 g/dL^31^.

The results of these and other experimental studies, however, are not easily extrapolated to clinical situations for patients with co-morbidities and changes in the balance of supply and demand of oxygen.

## Bases for the decision

Currently, "the transfusion threshold" is based on predetermined values for Hb concentrations that are derived from a few randomized clinically controlled trials, various observational cohort studies, or from the opinion of experts ^33^ ^,^ ^34^. This implies the existence of a Hb threshold level below which the transfusion should be initiated; a threshold that remains uncertain with current testing methods and the analysis of multiple observational studies and a few randomized controlled trials (RCTs)^35^. Unfortunately, the decision is also influenced by regulations, fear of future litigation, and public expectations more than clinical evidence^36^.

Two concepts form the basis for the use of the Hb concentration as a determinant of RBCT: the optimal level of Hb and the minimum acceptable level of Hb. The optimal level of Hb is the concentration of Hb at which organic functionality is maximal. Studies carried out on individuals undergoing acute normovolemichemodilution, found that oxygen transport (TO_2_) reaches a maximum at a 30% Hct (Hb 100 g/L), and it decreases as hemodilution and hemoconcentration progress. In diverse experimental animal models it has been shown that TO_2 _and survival are optimal at an Hct between 30 to 40%.

The minimum acceptable level of Hb is that point at which coronary blood flow cannot increase sufficiently to meet the oxygen demands of the myocardium. The minimum acceptable level of Hb should be considered as the transfusion threshold, but this level has yet to be clearly defined. While individuals with cardio-respiratory disease may need to maintain Hb levels >90-100 g/L to prevent signs of myocardial ischemia, healthy individuals with normal compensatory mechanisms can tolerate chronic levels of Hb from 50-60 g/L that maintains the blood volume.

That is, one should try to optimize the cardio-pulmonary hemodynamic in patients before making the decision to transfuse. In many of them an improvement in cardiac dynamics and supra-maximal O_2_ delivery can be achieved by increasing the concentration of inspired O_2_, correction of blood volume, the postoperative treatment of pain, etc.

Using the estimated volume of bleeding as a determinant of RBCT has two drawbacks: on the one hand, it is often difficult to determine and, in fact, at least in specific surgeries, the actual blood loss can be almost double that observed^37^. Also, the effects of bleeding will depend on factors such as the previous Hb, the circulating volume for the individual (which, in turn, depends on weight, height and sex), the rate of bleeding or the quality of volume replacement.

However, given that in the evaluation of the effects of anemia from acute blood loss are the critical factors of volume and rate of blood loss, as well as the degrees of hemodynamic instability. These all may be indicative of the need to transfuse. When losses are very rapid, in spite of volume replacement and hemodynamic stabilization of the patient, RBCT will very likely be needed to restore the O_2_ transport capacity, particularly in cases of severe trauma. Similarly, if the volume of bleeding is less than 25% of volume RBCT may rarely be necessary; between 25% and 50% may be frequently required, while acute loss of over 50% is almost always fatal.

The utility of the metabolic markers of hypoxia as determinants of RBCT is also limited. Lactate is produced in many hypoxic tissues as the end product of glycolysis under anaerobic conditions with peak plasma concentrations >2 mEq/L. However, the use of this metabolite as the only marker of tissue hypoxia, and therefore as a determinant for RBCT shows serious limitations from being influenced by the circulatory state, liver function, or concomitant sepsis. Thus, in a group of individuals under forty years of age, who are conscious and at rest and without cardiovascular, lung or liver disease, non-smokers who not taking medication effecting cardiovascular function, a decreased Hb level up to 50 g/L from acute normovolemichemodilution did not result in an inadequate O_2_ transport to the tissues as there was no change in either O_2_ consumption or plasma lactate concentration^30^.

On the other hand, the results of this study are consistent with those obtained in patients with acute myocardial infarction or sepsis in which no correlation was found between the levels of lactate and oxygen supply. Moreover, in any case, such a correlation would only indicate an overall change in oxygen supply but would not provide specific information on regional hypoxia.

The arterio-venous difference in the partial pressure of CO_2 _(P_a-v _CO_2_) can be a useful but nonspecific parameter for determining the presence of tissue hypoxia, especially in the post-operative setting of certain surgeries. After coronary artery bypass surgery, the P_a-v_CO_2_ is influenced by the metabolic rate, body temperature (possibly due to the release of CO_2_ during re-warming the patient) and decreased pulmonary elimination of CO_2_. In these circumstances, patients with abnormally elevated P_a-v_CO_2_ show a greater incidence of post-operative complications from tissue hypoxia (low cardiac output, arrhythmias, prolonged extubation, increased blood creatinine, jaundice)^38^.

The oxygen extraction quotient (CEO_2_) is the relationship between the consumption and delivery of O_2_ (VO_2_/DO_2_). It is expressed in percentages and indicates the percentage of O_2_ provided that has been utilized. Normal CEO_2_ is 25%. Some studies of normovolemic anemia performed with different animals have focused on the study of CEO_2_ as a good indicator of when to perform the T. Its application to daily clinical practice is lacking.

Although the only actual reason for establishing an indication for RBCT is for maintaining the O_2_ transport capacity, it does not always have the means to determine the transport capacity of O_2_ (e.g., SvO_2_, TO_2_, VO_2_, gastric pHi, blood lactate) and it is very difficult to know the time at which each patient needs to increase their transport capacity. This is especially so when you consider that their oxygen needs pre-operatively, during the intervention, and post-operatively are quite different.

Once anesthetized, the patient is in a state of minimal metabolic demand. If young, healthy individuals get adequate TO_2_ with Hb levels around 50 g/L, this same number should be applicable to individuals ASA I anaesthetized in which O_2_ consumption is lower than while at rest. Overall, general anesthesia with neuromuscular blockage and mechanical ventilation decreases oxygen consumption by 20-40%, which can make the critical TO_2_ descend to a lower level than in the conscious individual^39^.

In contrast, O_2_ demands increase during the post-anesthetic recovery and VO_2_ multiplies by a factor of 2-3, depending on the type of anesthesia used, the aggressiveness of the surgery, the degree of post-operative analgesia in the presence of pain and characteristics of the patient. Again, this situation, which is generally well-tolerated by young and healthy patients with active compensating mechanisms for anemia, may be post-operatively complicated by a compromised cardiovascular function, or among patients with sepsis, especially if they are elderly.A recent review^40^ concludes that due to the tremendous existing variability among patients with respect to delivery and extraction of O_2_ and cardiac reserve, the critical level of Hb has an individual value as there is no general threshold for RBCT. Given the evidence that myocardial ischemia is a critical factor in the patient's ability to tolerate anemia, no patient over forty years of age with Hb<100 g/L should undergo elective surgery without previously ruling out myocardial ischemia.

The effectiveness of the RBCT can only be established by results from well-designed randomized clinical trials. Until now the clinical trials conducted to compare two transfusion strategies ("restrictive" and "liberal") in different types of patients have not shown significant differences in terms of morbidity, mortality and functional status of patients, with the possible exception of those with AMI or unstable angina.

## Transfusion criteria

As previously noted, at the 1988 Consensus Conference organized by the U.S. National Institute of Health the threshold concentration of hemoglobin (Hb) from consensus was set at 70 g/L. It was emphasized that there was a direct call to establish needs and clinical symptoms as the basis for the transfusion decision, and not base the decision solely on Hb concentrations. That is, to transfuse if Hb<70 g/L, to individualize the decision for Hb levels of 70-100 g/L and not to transfuse if Hb>100 g/L. Since then, several guidelines have been published, the result of other Consensus Conferences along the same lines; that is, the reasons for the use of "restrictive" transfusion criteria prevailed over those more "liberal".

In the field of intensive care, in a multi-centric, randomized, prospective study, Transfusion Requirements in Critical Care (TRICC), the mortality rate in critically ill patients undergoing a "restrictive" RBCC protocol (Hb<70 g/L to keep it between 70 and 90 g/L) was compared with "liberal" criteria (Hb<100 g/L, to keep it between 100 and 120 g/L)^5^. The results of this study indicated that there was no difference in mortality when the two subgroups of patients with significant cardiac disease were compared.

However, according to the subsequent analysis of the TRICC study data^41^ variations in mortality rate after thirty days were found and opposite in the "liberal" group when compared to the mortality rate in the "restrictive" group, according to the presence or absence of coronary artery disease before randomization.

In subjects with ischemic heart disease, mortality was greater in the restrictive group than in the liberal group (26% versus 21%, respectively); whereas in patients without ischemic heart disease, mortality was lower in the restrictive group than in the liberal group (16% versus 25%, respectively) (Breslow-Day test, *p*= 0.03).

This analysis seems to demonstrate that the results of TRICC may be strongly influenced by the presence of non-comparable groups with different transfusion practices and are inadequate in each study group. The excess risk incurred by each of these subgroups makes comparing global mortality rates between the two transfusion strategies studied hard to interpret.

Given that the studies published before the TRICC indicated that clinicians used higher transfusion thresholds in patients with ischemic heart disease than in younger subjects with less co-morbidity, none of the study groups represents the usual practice.

In euvolemic surgical patients, almost all randomized studies to date have shown that the use of a "restrictive" transfusion threshold does not cause an increase in mortality or morbidity or in the duration of the hospital stay, while reducing both the percentage of patients transfused and the volume of allogeneic blood administered^42^ ^-^ ^48^ ^.^

The exception is from the work of Foss *et al*., but it was not designed to evaluate this objective and it lacked the statistical power to evaluate it^49^.

The recommendations of the AABB formulated in accord with the criteria of the GRADE methodology (Grades of Recommendation, Assessment, Development, and Evaluation), recently published are as follows^50^: 

Recommendation 1: The AABB recommends adhering to a restrictive transfusion strategy (7-8 g/dL) in hospitalized, stable patients (GRADE 1A: strong recommendation, high quality evidence).

Recommendation 2: The AABB suggests adhering to a restrictive strategy in hospitalized patients with a preexisting cardiovascular disease and consider transfusion in patients with symptoms or a hemoglobin of 8 g/dL or less (GRADE 2B: weak recommendation, moderate quality evidence).

Recommendation 3: The AABB cannot make a recommendation for or against the use of a liberal transfusion threshold or a restrictive one, for hospitalized patients, hemodynamically stable with acute coronary syndrome (GRADE 0: unclear recommendation, very low quality evidence).

Recommendation 4: The AABB suggests that transfusion decisions be based on the symptoms, as well as on hemoglobin concentrations (GRADE 2C: Weak recommendation, low quality evidence).

## Conclusions and recommendations

Reed blood cells transfuctions is widely used in the treatment of anemia, although adequate thresholds for its use remain controversial. Although therapeutic modalities should be subject to a rigorous evaluation of its efficacy and safety prior to use in clinical practice, RBCT has not been subjected to a similar examination. Besides the already known complications from transfusions, numerous studies indicate that RBCT may be associated with unfavorable outcomes.

The few conclusive results and non-controversial data have not overcome the difficulties that have prevented previous attempts to establish a policy or guide for RBCT. Despite these limitations and the lack of definitive answers, doctors often have no other choice but the consensus guidelines^34^.

In building a "standard of care", consensus guidelines and reviews often do not represent the actual current clinical practice, and several examples illustrate its ineffectiveness in modifying clinical practice^51^ ^-^ ^54^ ^.^

Almost two thirds of physicians report regularly transfusing RBC in probably unnecessary situations,^55^and there is wide variation in transfusion practices with respect to weight that is attributed to clinical factors used in decision making, which also hinders the characterization of current practice^56^.A high priority for future clinical investigations should be determining the efficacy of RBCT in those situations classified as uncertain. In the absence of data, it is prudent that RBCT be administered with caution in these clinical scenarios. Therefore, we should do it on an individual basis, i.e., carefully weighing the risks of anemia and the risks and benefits to be derived from each of these products for each patient, supporting them with the proper dosage and monitoring the expected therapeutic response, simultaneously with the application of an appropriate alternative to RBCT and efficient for our patient at all times. That is, we must seek the maximum benefit with the least possible exposure.
